# Intraoperative Fluoroscopic Radiation Exposure During Hip Fracture Fixation: A Study Combining Surgical Experience and Fracture Complexity

**DOI:** 10.7759/cureus.17393

**Published:** 2021-08-23

**Authors:** Angus Bruce, Chiranjit De, Ramez Golmohamad, Awais Habeebullah, Manoj Sikand, Aashish Gulati

**Affiliations:** 1 Department of Trauma & Orthopaedics, Sandwell and West Birmingham NHS Trust, Birmingham, GBR; 2 Department of Trauma & Orthopaedics, Epsom and St Helier University Hospitals NHS Trust, London, GBR; 3 Orthopaedics, Royal Orthopaedic Hospital, Birmingham, GBR

**Keywords:** ionising radiation, radiation exposure, hip fractures, surgical experience, complexity

## Abstract

Aim

Hip fracture fixation surgeries are one of the most common surgeries that every trauma unit does regularly. Surgical training and expertise to fix these fractures properly are quite crucial for every orthopaedic surgeon. Therefore, orthopaedic training programmes all over the world consider significant focus on this and teach trainee surgeons expectantly to manage these fractures independently. Surgical fixation of hip fractures often requires fluoroscopy assistance in the operating theatre with associated hazards from ionising radiation. Moreover, hip fractures can be sometimes quite complex and may require relatively more fluoroscopy usage even with the higher grade of the operating surgeons. Therefore, training need for hip fracture fixation surgery is imperative and there is also a need for intraoperative radiation safety. This study has tried to find a balance between intraoperative fluoroscopic radiation exposure, surgical training requirement, and hip fracture complexity.

Methodology

This single centre study has collected retrospective peri-operative data over a period of two years including hip fractures that required fluoroscopy-guided surgical fixation. Femoral head fractures, subtrochanteric fractures, diaphyseal fractures, and trochanteric fractures with associated pelvic fractures were excluded from the study. We collected data on demographic parameters, fracture complexity and grading (Arbeitsgemeinschaft für Osteosynthesefragen/Orthopaedic Trauma Association [AO/OTA] Classification), intraoperative ionising radiation exposure (centi-Gray/cm^2^), and grade of the operating surgeon in order to find any relation between these factors.

Results

Total 268 patients were included in the study with a mean age of 81.8 years (SD 9.3) comprising of 83 (31%) male patients and 185 (69%) female patients. The study population was further stratified into three groups depending upon the operating grade of the surgeon: ‘Junior Trainee’ (<five years of experience; 77 cases [29%]); ‘Senior Trainee’ (>five years of experience; 148 cases [55%]); and ‘Consultant’ (fully trained to practice independently; 43 cases [16%]). There was no statistically significant difference among these three sub-groups with regards to the age (p = 0.79), gender (p = 0.73), body mass index (p = 0.46), and fracture pattern (p = 0.96) of the patients. However, consultants tend to operate more on the higher American Society of Anesthesiologists (ASA) grade patients (p = 0.049) with more comorbidities. There was statistically significant higher fluoroscopic radiation exposure while junior trainee surgeons (p = 0.005) were operating and during the higher complex grade of hip fracture (p = <0.001) fixation.

Conclusion

In conclusion, the quantity of intra-operative radiation dose utilised in the surgical fixation of hip fractures is significantly associated with the grade and level of training of the operating surgeon and fracture complexity type. The results of this study emphasise and support the importance of comprehensive, supervised, and structured orthopaedic training for in-theatre radiation safety. It is recommended to have a safe balance between teaching, learning, and prevention of ionising radiation hazards in order to optimally achieve trainee’s professional development with successful patient outcomes.

## Introduction

Modern orthopaedic practice heavily relies on the appropriate use of fluoroscopic imaging. During the surgical fixation of hip fractures, intra-operative imaging provides a valuable guide to assess the quality of fracture reduction, while also ensuring that the final implant position is of a reasonable standard. However, exposure to prolonged ionising radiation has proven associations to various harmful effects. There is evidence suggesting that orthopaedic surgeons are at greater risk of the development of malignancy, a possible consequence of increased occupational exposure to ionising radiation [1,2]. An association has also been identified between occupational radiation exposure and male infertility, as well as the development of cataracts [3,4].

Around 70,000-75,000 hip fractures occur annually in the United Kingdom [5], with numbers only expected to rise as a direct result of an ageing population. These demographic changes have resulted in the projection of as many as 6.26 million hip fractures occurring annually worldwide by the year 2050 [6]. As such, many orthopaedic surgeons learn to perform hip fracture surgery during the early years of their training. The Joint Committee on Surgical Training (JCST) require evidence of a minimum of 40 performed compression hip screw for the intertrochanteric neck of femur fracture, for certification following 72 months of orthopaedic training [7]. There is no doubt that a high-quality surgical fixation leads to better post-operative patient outcomes [8-10], and the use of fluoroscopic imaging helps to facilitate this. While it is important to ‘Get it right first time’ (GIRFT) [11] and ensure that key steps of the surgery are performed to a very high standard irrespective of the time (and radiation spent), a delicate balance must be struck between GIRFT and the optimisation of radiation exposure.

Few available studies have explored the association between the surgeon grade, modality of fixation, and the intra-operative radiation dose used in the surgical fixation of hip fractures. However, there is limited evidence correlating the surgeon grade, fracture severity, and radiation exposure altogether. The primary aim of this study was to explore whether there is any relationship between these variables. The secondary aim was to identify any associated factors that have contributed to increased radiation used during surgery. Our ambition was for our results to act as a guide in order to suggest measures to reduce intra-operative radiation exposure for surgeons and patients alike.

## Materials and methods

Study design

This study was performed at a large district general hospital in the West Midlands, United Kingdom, covering a catchment area of over 500,000 people. Data were collected over a two-year period, from January 2018 to December 2019. All the hip fractures fixed during this time using intraoperative fluoroscope were included in the study. Electronic patient records, operative notes, anaesthetic checklists, and radiographs were used to collate and analyse data. Data regarding the radiation dosage was provided from records made immediately post-operatively by the radiographer present in the operating theatre. The study provided level III evidence.

During the specified period, 268 patients with extracapsular hip fractures required surgical fixation. The fractures were classified according to the Arbeitsgemeinschaft für Osteosynthesefragen/Orthopaedic Trauma Association (AO/OTA) classification model [12]. The classification model provides subdivision of all trochanteric fractures, defined as any fracture centred below the intertrochanteric line and above the horizontal transverse line at the inferior border of the lesser trochanter. A1 fractures are defined as simple per-trochanteric fractures; A2 as multifragmentary per-trochanteric, lateral wall incompetent (<20.5 mm) fractures; and A3 as intertrochanteric (reverse obliquity) fractures. Femoral head fractures, subtrochanteric fractures, diaphyseal fractures, and trochanteric fractures with associated pelvic fractures were excluded from the study.

All patients who had a surgical fixation for the neck of femur fracture and the recording of the radiation dosage availability from the radiographers were included. Fractures requiring open reduction were excluded because of the complexity, and hence the additional radiation involved. The grade of the operating surgeon was subdivided into a ‘Consultant’ (fully qualified with a certificate of training and allowed to practice independently), ‘Senior Trainee’ (>five years of experience in trauma and orthopaedics), and ‘Junior Trainee’ (<five years of experience). Radiation dosage was measured by centi-Gray/cm^2^ (cG/cm^2^).

Statistical analysis

Statistical analysis was performed using SPSS Statistics version 22.0 (IBM SPSS Statistics, Armonk, NY). A two-way analysis of variance was applied to determine the significance of surgeon grade and fracture type to the dose of radiation. Tukey’s post hoc tests were performed to perform sub-group analysis. A p-value of <0.05 was considered statistically significant.

## Results

Cohort characteristics

From January 2018 to December 2019, 305 patients had surgical fixation of neck of femur fracture, of which 268 met inclusion criteria. A summary of the baseline patient demographics and fracture complexity can be found in Table 1. The primary operating surgeon was a consultant in 43 cases (16%), senior trainee in 148 cases (55%), and junior trainee in 77 cases (29%). There was no statistically significant difference in age (p = 0.79), body mass index (p = 0.46), or gender (p = 0.73) within the groups operated on by consultants, senior trainees, and junior trainees.

**Table 1:** Demographic profile of the study population (n = 268). SD - standard deviation, BMI - body mass index, M - male, F - female, ASA - American Society of Anesthesiologists, AO/OTA - Arbeitsgemeinschaft für Osteosynthesefragen/Orthopaedic Trauma Association.

Variables	Consultant (n = 43)	Senior Trainee (n = 148)	Junior Trainee (n = 77)	Total (n = 268)	p-value
Age (years) (Mean ± SD)	82.9 ± 9.8	81.3 ± 9.0	81.9 ± 9.5	81.8 ± 9.3	0.79
BMI (Mean ± SD)	26.1 ± 7.1	25.3 ± 5.0	26.2 ± 6.3	23.1 ± 5.9	0.46
Gender (M:F)	14:29	49:99	21:56	83:185	0.73
ASA grade (%)					
ASA-1	0	2	3.9	2.2	0.049
ASA-2	9.3	25	22.1	21.6	
ASA-3	53.5	58.8	61	58.6	
ASA-4	37.2	14.2	13	17.6	
Fracture classification (AO/OTA)					
A1	19	76	38	133	0.96
A2	19	59	35	113	
A3	5	13	4	22	

When analysed by the American Society of Anesthesiologists' (ASA) classification, there was a significant difference between the three groups (p = 0.049), with consultants tending to operate on patients with a higher ASA grade and higher comorbidities. However, there was no statistically significant difference in the grade of the operating surgeon and the fracture complexity (p > 0.05). This could have given relatively unbiased values of radiation exposure due to the skill mix while fixing different grades of hip fracture.

Correlation of radiation dosage as per surgeon grade

Table 2 demonstrates the mean intra-operative radiation dose when stratified for consultants, senior trainees, and junior trainees. There was a statistically significant difference in the amount of radiation exposure between the three groups (p = 0.005) indicating that junior trainees were involved in higher radiation exposure.

**Table 2:** Radiation dosage as per surgeon’s grade. cG/cm^2 ^- centi-Gray/centimeter^2^, SD - standard deviation.

	Consultant	Senior trainee	Junior trainee	p-value
Intra-operative radiation dose (cG/cm^2^) (Mean ± SD)	203.2 ± 17.8	177.6 ± 22.5	261.3 ± 16.8	0.005

A sub-group analysis (Table 3) demonstrated that the senior trainees used a significantly lower radiation dose compared to the junior trainees (p = 0.005). There was, however, no statistically significant difference in the radiation dosage used by the consultants and the senior trainees (p = 0.537), or between consultants and junior trainees (p = 0.341).

**Table 3:** Radiation dosage comparing sub-group analysis for surgeon’s grade. Statistical test: Tukey’s post hoc test.

Sub-group analysis	p-value
Consultant vs senior trainee	0.537
Consultant vs junior trainee	0.341
Senior trainee vs junior trainee	0.005

Correlation of radiation dosage as per fracture type

When categorised according to AO/OTA [12] classification of the fracture type, there was a significant association of increasing fracture severity with increasing intra-operative radiation dose (p < 0.001). Table 4 displays the radiation dosage used classified by the fracture type.

**Table 4:** Radiation dosage as per fracture type. cG/cm^2 ^- centi-Gray/centimeter^2^, SD - standard deviation.

	A1	A2	A3	p-value
Intra-operative radiation dose (cG/cm^2^) (Mean ± SD)	152.3 ± 13.2	222.7 ± 18.4	343.3 ± 37.8	<0.001

Table 5 summarises a sub-group analysis between each fracture type. It is noticeable that for every higher fracture grade, there is a statistically significant increase in radiation exposure (p < 0.05). Overall, these two tables summarise statistically significant higher intra-operative radiation exposure with regards to higher fracture complexity.

**Table 5:** Radiation dosage comparing sub-group analysis for different fracture patterns. A1, A2, A3 - Arbeitsgemeinschaft für Osteosynthesefragen/Orthopaedic Trauma Association (AO/OTA) extracapsular hip fracture classification. Statistical test - Tukey's post hoc test.

Fracture pattern sub-group analysis	p-value
A1 vs A2	0.003
A2 vs A3	0.005
A1 vs A3	<0.001

## Discussion

Our study demonstrates that the amount of intra-operative radiation dose used in the fixation of hip fractures is highest when patients are operated upon by the junior trainees. The data analysis showed that the junior trainees used nearly 50% higher radiation dose when compared to the more senior trainees. As with the progression of the learning curve, the radiation exposure during hip fracture fixation gradually reduced. Botchu et al. [13] performed a similar study which showed that the radiation exposure and the fluoroscopy screening time were significantly high for trainees under three years of surgical experience. However, their comparator group consisted of surgeons with more than 10 years of experience. In comparison, we have tried to report on a stratified cohort of three groups to provide stronger evidence. Unlike their study which reported on the two extremes of the learning curve, we have tried to sub-divide the trainee group further to assess the implication of learned skills upon the radiation dosage. Indeed, the experience of the operating surgeon helped to reduce the radiation exposure providing supportive evidence for the trainee’s evolving expertise as they climb the learning curve. Moreover, the sub-group analysis further has provided supportive evidence for learning progression as a factor of statistically significant difference in radiation exposure and screening time. Though the amount of radiation used by consultants was higher than senior trainees (14.4%), this was not statistically significant. This could be hypothesised that the consultants intended to operate on patients with comparatively complex fracture patterns. Due to the inherent nature of complexity in those fixation surgeries, it might have incurred higher radiation exposure.

On the contrary, Quah et al. [14] found that the registrar cohort as a whole (ST3-ST8), cumulatively used a higher dosage of ionising radiation compared to the consultant cohort for an extended series of 1203 patients. It is notable that this study again compared two extremes of surgical expertise. It is a fact that trauma surgery follows the curve of progressive professional development and, at times, it could be quite steep and tedious. However, Quah et al. have shown in their sub-group analysis that the senior trainees used lesser fluoroscopy screening time and radiation compared to the junior trainees. These findings are again parallel with our sub-group analysis and which again supports the learning progression.

Interestingly, in a series of 137 hip fracture patients, Kelly et al. [15] studied multiple factors guiding the ionising radiation exposure for hip fracture fixation and found no statistically significant difference in fluoroscopy time used by the registrars and consultants. However, this study did not sub-classified the hip fractures, rather concentrated upon the mode of fixation. In comparison, we reported with a sub-group analysis with regards to fracture type and the experience of operating surgeons. In a sense, our study has tried to match the fracture complexity and surgical expertise for a succinct outcome. The study shows that the A3 sub-group (AO/OTA Classification) consumed more radiation compared to other fractures. However, surgeon-matched sub-group analysis finds no statistically significant difference in radiation exposure for the fixation surgeries in different patterns. This further supports the hypothesis that with progressive years of training, senior trainees fix the more complex fractures compared to the junior trainees with considerable radiation exposure. However, there could be a type-II error due to variable complexity within a specific fracture sub-group due to peri-operative parameters. This might be the possible reason for consultants ending up operating on more complex fractures with higher radiation exposure. Overall, it is noticeable that the radiation dosage for hip fracture fixation follows the learning curve of a trainee surgeon and also supports that with progressive development they are able to fix more complex fractures safely.

Rashid et al. [16] studied 849 cases of orthopaedic trauma surgery with a maximum number of proximal femoral fracture surgeries that required fluoroscopy assistance showing significantly reduced fluoroscopy exposure with increasing surgeon’s experience. This is indeed the responsibility of the surgeon to ensure the radiation safety of the operating room personnel. However, the training needs of the surgical trainees cannot be deferred as well. Therefore, a fine balance is always needed for safe learning. The result of our study can support this idea with evidence of senior trainees using lesser radiation dosage.

Buxbaum et al. [17] conducted almost a similar study on 852 proximal femur fractures to interpret the correlation between the level of resident training and radiation exposure during fixation. They also reported relatively lower radiation exposure with surgeons with a higher level of resident training. However, they included sub-trochanteric fractures in their study population. The complexity of sub-trochanteric fractures is often varied and sometimes challenging too. This was the only subtle difference they had from our study population. Overall, the results of both studies can be extrapolated to a close conclusion. There is also consensus that more senior surgeons should take an active role to reduce radiation hazards.

Riaz et al. [18] have tried to develop a model which is predictive and reproducible to minimize the radiation exposure to the patient and the staff during trauma surgery. They have reiterated the experience of the surgeon as one of the prime factors. In our study, we have successfully reproduced this fact with proven results. Moreover, in the current national training structure, the role of the supervising consultant is also pivotal for in-theatre radiation safety. Therefore, the result of this study supports the fine-tuning of teaching, learning, and ionising radiation safety add immense value towards the progression curve of the orthopaedic trainee.

Our results support the belief that the additional experience and confidence associated with seniority aids the decreased use of potentially harmful ionising radiation. It would be appropriate to suggest that this is associated with increased experience and skill during reduction manoeuvres, correct guidewire positioning with minimum attempts, experience with the judgement of screw size, and metalwork positioning. Indeed, surgical expertise is a learned skill and every trainee has to climb the learning curve. The findings from this study emphasise the importance of comprehensive and structured orthopaedic training. Furthermore, teaching and training in a ‘radiation safe environment’ remain the responsibility of the trainer and the trainee both. It is very difficult to draw a line when a junior trainee becomes a senior trainee, and the learning curve resolves. However, the national training structure has been designed to it best to support the learning in a safe environment.

It is, however, mandatory that the fixation quality should not be compromised, even when making a concerted effort to reduce intra-operative radiation dosage. For that reason, it is recommended that a consultant always scrubs/supervise the surgery on patients with complex hip fractures, so as to provide experienced support to the trainee, helping achieve high-quality fixation, while reducing the amount of radiation administered to the patients and also to the operating room personnel.

We acknowledge the limitations of a single centre, retrospective study with limited sample size. Nevertheless, this paper provides important information for the relationship between the seniority of a surgeon, the complexity of the hip fracture surgery, and the radiation exposure from the use of intra-operative fluoroscopic imaging. It is acknowledged that there will be some variation amongst individual surgeons, even if they are fully trained, as they will have different skills and steps that require the use of intra-operative imaging. However, this difference could be minimum because of the standard training structure. A larger multi-centre study involving even greater numbers of surgeons of varying experience will help reduce the effect of this confounding factor. Importantly, the experience of the radiographer is also crucial, with the assumption made those radiographers of experience will orient themselves quicker and require fewer initial radiographs to find the anatomical landmarks.

## Conclusions

In conclusion, the quantity of intra-operative radiation dose utilised for surgical fixation of hip fractures is significantly associated with the grade and level of training of the operating surgeon and fracture severity type. The results of this study emphasise and support the importance of comprehensive, supervised, and structured orthopaedic training for in-theatre fluoroscopic radiation safety. It is recommended to have a safe balance between teaching, learning, and prevention of ionising radiation hazards in order to optimally achieve trainee’s professional development with successful patient outcomes.
